# Combination of Metronomic Chemotherapy and Rituximab in Frail and Elderly Patients with Relapsed/Refractory Follicular Lymphoma and Ineligible for Lenalidomide Treatment: A Retrospective Analysis

**DOI:** 10.3390/cancers18020347

**Published:** 2026-01-22

**Authors:** Sabrina Pelliccia, Marta Banchi, Lucrezia De Marchi, Emanuele Cencini, Claudia Seimonte, Alberto Fabbri, Andrea Nunzi, Susanna Destefano, Guido Bocci, Maria Christina Cox

**Affiliations:** 1 Haematology Unit, Azienda Ospedaliera Universitaria Sant’Andrea, 00189 Rome, Italy; 2 Department of Translational Research and New Technologies in Medicine and Surgery, School of Medicine, University of Pisa, 56126 Pisa, Italy; 3 Department of Biomedicine and Prevention, University Tor Vergata, 00133 Rome, Italy; 4 Haematology Unit, Azienda Ospedaliera Universitaria Senese, 53100 Siena, Italy; 5 Clinical Pathology, University Tor Vergata, 00133 Rome, Italy; 6 Hematology Unit, Fondazione Policlinico Tor Vergata, 00133 Rome, Italy

**Keywords:** follicular lymphoma, relapsed/refractory, metronomic chemotherapy, rituximab, elderly, frail, lenalidomide-ineligible

## Abstract

Background/Objectives: Relapsed or refractory follicular lymphoma (rrFL) remains difficult to treat in elderly or frail patients who cannot tolerate standard-dose immuno-chemotherapy as well as novel therapies. Metronomic chemotherapy (mCHEMO) may offer sustained antitumor activity with reduced toxicity. This study assessed the clinical activity and safety of R-DEVEC or R-DEVEC-light in rrFL patients following lenalidomide discontinuation or ineligibility. Methods: Data from the ReLLi Lymphoma Registry (2013–2025) were retrospectively analyzed. Eligible patients had rrFL after ≥1 prior therapy and initiated mCHEMO at least six months before data cutoff. Thirteen patients received DEVEC or the etoposide-free DEVEC-light regimen; all but one also received rituximab. Responders received maintenance vinorelbine, low-dose prednisone, and rituximab, followed by vinorelbine-only maintenance until progression or intolerance. Responses were assessed by CT after cycle two and PET/CT at completion of six induction cycles. Results: median age was 77 years (range 58–92); most patients were frail and had advanced disease. At the end of induction, 84% achieved remission (46% CR, 38% PR), with three PR converting to CR during maintenance. After a median follow-up of 27 months, the PFS was 42% (95CI 15–69%) and the OS 73% (95CI 47–100%). A transformation occurred in one patient; the main toxicity was grade 3 neutropenia (31%). DEVEC-light showed improved tolerability versus full DEVEC, with manageable infections and rare discontinuations. Conclusions: Metronomic R-DEVEC-light is a feasible and effective disease-controlling strategy for frail, heavily pretreated rrFL patients who do not tolerate lenalidomide and are excluded from modern therapies. This schedule warrants further prospective evaluation and exploration in combination with targeted agents.

## Simple Summary

Relapsed or refractory follicular lymphoma (rrFL) is challenging to treat, especially in elderly and frail patients who often cannot tolerate therapies such as standard-dose immuno-chemotherapy, lenalidomide-based regimens, or CAR-T cell therapies. This study evaluates the metronomic, all-oral R-DEVEC or R-DEVEC-light regimen as a feasible, low-toxicity alternative for this vulnerable population. We aim to retrospectively assess the regimen’s real-world activity and safety in patients who discontinued or were ineligible for lenalidomide therapy. R-DEVEC-light show high response rates, manageable toxicity, and prolonged remissions, suggesting that metronomic chemotherapy may stabilize disease without compromising sensitivity to future treatments. If validated in larger studies, this approach could meaningfully expand treatment options for frail rrFL patients, offering a cost-effective, home-based therapy that fills an important gap in the current therapeutic landscape.

## 1. Introduction

Follicular lymphoma (FL) is the most frequent indolent non-Hodgkin lymphoma (NHL), accounting for about 5% of all hematological neoplasms and about 20–25% of all new NHL cases in Western countries [1]. It is more prevalent in older adults, with a median age at diagnosis of 60–65 years, and its incidence increases progressively from around 35 years of age, reaching a peak at approximately 70 years. Although its natural history is typically indolent, FL is still considered incurable and characterized by multiple relapses. This is a heterogeneous disease, with some patients exhibiting early disease progression, histologic transformation, or a significant risk of treatment-related toxicity [2,3,4,5]. Treatment decisions in relapsed/refractory FL (rrFL) are complex and must consider prior therapies, disease biology, as well as patient age, comorbidities, individual preferences, and treatment accessibility [3]. Moreover, early relapse within 24 months of first-line treatment (i.e., POD24), which occurs in approximately 20% of patients [6], and histologic transformation into a more aggressive subtype, typically diffuse large B-cell lymphoma (DLBCL) [7], are associated with poor prognosis and limited therapeutic success with immuno-chemotherapy [8].

For rrFL, lenalidomide combined with anti-CD20 antibody (i.e., rituximab and obinutuzumab) and, more recently, with the addition of tafasitamab, is an established approach [5,9,10]. Novel agents such as bispecific antibodies, chimeric antigen receptor (CAR) T-cell therapies, and epigenetic modulators have shown unprecedented efficacy in poor prognosis rrFL [11]; however, these options may be inaccessible or unsuitable for elderly or frail subjects due to toxicity, logistical constraints, or cost [12]. Metronomic chemotherapy (mCHEMO)—the frequent and regular oral administration of cytotoxic agents that can maintain low, prolonged, and active plasma levels of drugs—represents an alternative strategy that allows a sustained antitumor effect with reduced toxicity [13]. mCHEMO has shown to act through multiple, distinct biological pathways. These include the following: (1) suppression of tumor angiogenesis, mediated by selective toxicity toward endothelial cells and the induction of angiogenesis-dependent tumor dormancy; (2) direct cytotoxic activity against cancer stem cells, tumor-initiating populations, and differentiated malignant cells; (3) modulation of the immune response through enhancement of cytotoxic T-cell activity via depletion of regulatory T-cells (Tregs) [14]. In haemato-oncology, a number of recently introduced agents—including IMIDs, azacytidine, and decitabine—have been classified as cytotoxic drugs. Notably, their therapeutic benefit is predominantly observed when they are delivered at low or metronomic doses. Furthermore, several long-established cytotoxic agents still exhibit pronounced anti-lymphoma efficacy when administered in a metronomic manner, reflecting their ability to interfere with diverse molecular and cellular targets [14].

Cyclophosphamide, a cornerstone alkylating agent in the treatment of NHL, is among the most extensively investigated examples. Its biological effects vary substantially depending on the systemic drug levels achieved through different metronomic dosing regimens [13]. In murine models, oral cyclophosphamide, administered continuously at low doses (20 mg/kg), has been shown to selectively impair endothelial cell proliferation and reduce tumor-associated microvascular density [15]. Conversely, intermittent metronomic dosing (140 mg/kg, intraperitoneally) every six days results in robust activation of innate immune effector cells, including macrophages, natural killer cells, and dendritic cells [16].

Vinorelbine, when delivered metronomically, has likewise been widely characterized as an inhibitor of angiogenesis across a range of in vitro and in vivo models, primarily through suppression of endothelial progenitor cell activity [17,18,19]. Importantly, vinorelbine used at metronomic concentrations (i.e., pico- to nanomolar ranges in vitro, or 3–6 mg/kg thrice weekly in vivo) also induces direct tumor cell cytotoxicity [20], significantly lowers circulating Treg numbers following prolonged treatment [21], and reduces plasma interleukin-2 levels [22]. Together, these effects correlate with marked antitumor efficacy.

Over the past ten years, mCHEMO has emerged as a promising treatment strategy for solid tumors [23,24,25,26]. Unfortunately, research on mCHEMO in aggressive lymphoma, both preclinical and especially clinical, is limited [14]. Notwithstanding, the outcomes are impressive and encourage the application of tolerable and effective mCHEMO regimens for advanced hematological malignancies, or at least for frail and elderly patients, who are not eligible or resistant to standard treatments [27]. Recently, we reported the efficacy of the all-oral DEVEC schedule (Deltacortene^®^ [prednisone], etoposide, vinorelbine, and cyclophosphamide) +/− rituximab for both treatment naïve and r/r elderly subjects, with B- and T-cell aggressive NHL, with acceptable toxicity [28,29,30]. A subsequent study confirmed its feasibility as first-line therapy in very elderly and/or frail diffuse large B-cell lymphoma (DLBCL) patients, with overall survival (OS) and disease-free survival at 24 months of 54% and 74%, respectively [31]. Given the unmet need for tolerable, affordable, and effective therapies in elderly and frail rrFL patients, an underrepresented group in clinical trials, we retrieved from the Lymphoma Registry of the Lazio Region (ReLLi) 13 patients with rrFL, who received R-DEVEC or its etoposide-free variant, R-DEVEC-light, due to discontinued lenalidomide for excessive toxicity, refractoriness, or unavailability of the drug. This study shows that the metronomic R-DEVEC-light may be a promising regimen for elderly rrFL in the evolving therapeutic landscape of non-Hodgkin lymphomas. Our regimen represents a feasible, low-intensity and home-based treatment capable of achieving meaningful and durable disease control in elderly rrFL patients who are excluded from standard-dose chemo-immunotherapy and current targeted approaches.

## 2. Patients and Methods

Data were retrieved from the ReLLi Lymphoma Registry (ethical approval no. 98172012, date of approval 26 November 2012; ethical approval no. 50.23, date of approval 6 June 2023). Thirteen eligible subjects met the following criteria: (a) relapsed or refractory FL after at least one prior line of therapy; discontinuation of lenalidomide due to toxicity, refractoriness, or lenalidomide unavailability at the time of treatment initiation; (b) signed written informed consent; (c) initiation of metronomic chemotherapy (mCHEMO) at least six months before the data cutoff of 31st August 2025.

Patients received either an all-oral DEVEC or DEVEC-light regimen, with or without rituximab. Rituximab (375 mg/m^2^) was administered weekly for four infusions, then monthly for an additional four doses. Six induction cycles were delivered following this schedule: cyclophosphamide 50 mg/day, up to 21 days; vinorelbine 30 mg, thrice weekly, 3 weeks on/1 week off; with or without etoposide, 50 mg/day, up to 14 days; prednisolone, 25 mg thrice weekly.

Patients achieving partial (PR) or complete response (CR) received maintenance therapy consisting of vinorelbine 30 mg, thrice weekly, 3 weeks on/1 week off; prednisolone, 12.5 mg twice weekly for six months; rituximab, bimonthly for up to eight cycles.

After completion of this maintenance phase, patients in PR or CR continued metronomic vinorelbine-only maintenance until disease progression or unacceptable toxicity.

Intermediate and final disease restaging were performed with a CT scan after cycle two and with CT-PET at completion of the induction phase, respectively. Toxicities were assessed and graded according to standard criteria.

## 3. Results

Between February 2014 and July 2024, thirteen patients initiated mCHEMO: three received DEVEC (pt # 1, 12, and 13 in the swimmer-plot) and 10 received DEVEC-light schedules. All but one patient received rituximab because of a previously developed serious rituximab-related adverse event. The median patient’s age was 77 years (range 58–92), where 11 patients had advanced-stage disease, and 10 were classified as frail based on comprehensive geriatric assessment (CGA) [32].

Six patients began mCHEMO after discontinuing lenalidomide due to toxicity; in three patients, lenalidomide was contraindicated because of severe kidney impairment (*n* = 1) and advanced-stage cirrhosis (*n* = 2); in three cases, it was unavailable at the time of treatment, while a last patient was refractory (Table 1).

### 3.1. Efficacy

**Figure 1:**
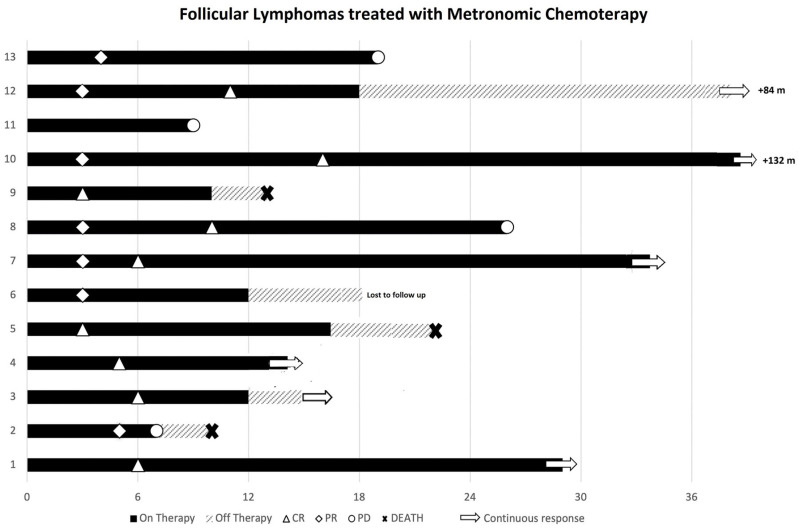
Treatment timelines of 13 patients with follicular lymphoma receiving metronomic chemotherapy.

At the end of induction, eleven patients achieved remission: six CR and five PR, with an overall response rate (ORR) of 84%. During maintenance, three PR patients converted to CR at 9, 10, and 16 months after mCHEMO initiation (Figure 1).

At the data cutoff (31 August 2025), six patients remained in remission, and two who relapsed achieved stable CR after treatment with lenalidomide–rituximab (R^2^) and obinutuzumab-CVP, respectively. One patient was lost to follow-up.

**Figure 2:**
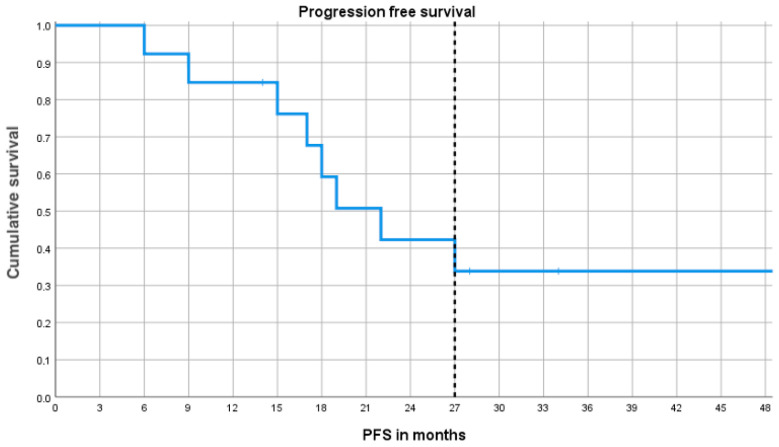
Kaplan–Meier curve of progression-free survival (PFS) in patients with follicular lymphoma treated with metronomic chemotherapy.

The median PFS was 22.1 months (95%CI 15.3–28.6), while at the median follow-up of 27 months, the progression-free survival (PFS) and OS were 42% (95CI 15–69%) and 73% (95CI 47–100%), respectively (Figure 2).

Four patients progressed: two were in PR, one was not responsive to mCHEMO, and the fourth was in CR. Two were treated with R^2^, and one with Obinutuzumab-CVP, and all of them achieved a durable CR. The fourth patient, who had a transformation to high-grade lymphoma, died within two months (Figure 1).

### 3.2. Safety

The main toxicity was grade ≥ 3 neutropenia (31%). Among the three, heavily pretreated patients treated with R-DEVEC, one developed febrile neutropenia, and two developed infectious complications (i.e., COVID-19 and bacterial pneumonia). All three were switched to DEVEC-light after a median of two cycles (range, 1–3). During R-DEVEC-light induction, four patients required occasional G-CSF administration for grade 3 neutropenia.

Eleven patients in remission at the end of induction began maintenance therapy.

After completing the six planned maintenance cycles, eight patients in PR or CR proceeded to vinorelbine-only maintenance. Three heavily pretreated individuals with substantial comorbidities discontinued vinorelbine due to grade 3 neutropenia (*n* = 2) or pneumonia (*n* = 1). At the cutoff date, three patients remained on vinorelbine-only maintenance (+18, +21, and +112 cycles).

At a median follow-up of 27 months, three deaths occurred, two of which occurred during treatment: one patient for cardiac failure following pneumonia (who previously suffered from significant heart disease and recurrent pulmonary infections) and one for FL transformation to high-grade lymphoma. One 94-year-old patient died six months after treatment stopped for infection-related multi-organ failure.

## 4. Discussion

This retrospective and registry-based analysis demonstrates that metronomic R-DEVEC or R-DEVEC-light schedules can achieve meaningful and durable clinical activity, with an acceptable safety profile, in frail and heavily pretreated patients with rrFL. Despite the high-risk features of this cohort, including advanced age, extensive comorbidities, prior lines of therapy, and frequent discontinuation of lenalidomide due to toxicity, our schedule induced an ORR of 84% at the end of induction, including 46% of patients achieving CR and 38% PR. More importantly, three patients in PR converted to CR during maintenance, suggesting that sustained and low-dose exposure to VNR may deepen responses over time.

These findings align with the multi-targeted pharmacological mechanisms characteristic of metronomic chemotherapy. In particular, the anti-angiogenic activity of metronomic vinorelbine (mVNR) may progressively disrupt tumor microvasculature and stromal support, leading to delayed but deepening tumor responses over time. In parallel, the immunomodulatory effects of mCHEMO—including the enhancement of effector CD8+ T-cell and NK-cell function, dendritic cell maturation, and depletion of immunosuppressive cellular populations (i.e., regulatory T-cells and myeloid-derived suppressor cells) [33], may contribute to sustained immune-mediated tumor control during maintenance, thereby facilitating PR-to-CR conversion.

Furthermore, the preserved sensitivity to subsequent therapies observed in our cohort supports the hypothesis that, unlike conventional high-dose administration, mVNR exerts anti-lymphoma activity primarily through its immunomodulatory and anti-angiogenic properties, rather than by directly selecting for chemotherapy-resistant lymphoma cells [13,33]. Notably, this mechanism provides a biological rationale for the lack of cross-resistance to subsequent lines of chemotherapy or lenalidomide observed in our patients following prior exposure to R-DEVEC-light. Moreover, it supports therapeutic sequencing strategies that alternate or integrate metronomic and standard-dose regimens over the disease course [34].

Lenalidomide has direct anti-tumor activity against activated B-cell-like (ABC) DLBCL cells by downregulating IRF4 expression and inhibiting B-cell receptor (BCR)–mediated NF-κB signaling pathway in a cereblon (CRBN)-dependent manner [35]. Resistance to lenalidomide in B-cell lymphomas is mainly driven by disruption of the CRBN–IRF4–NF-κB/IFN-β axis within tumor cells, as well as by impairment of dendritic cell maturation in the tumor microenvironment [36,37]. Interestingly, tafasitamab plus mVNR combination has recently shown to inhibit the phosphorylation of most proteins involved in the BCR-mediated PI3K/Akt/mTOR signaling pathway in DLBCL cells [20]. In addition, in sensitive ABC-DLBCL lines, the inhibition of PI3K/Akt/mTOR using a PI3Kβ/δ inhibitor decreased NF-κB activity and NF-κB target genes, including IRF4 [38]. Taken together, these observations suggest that suppression of this survival pathway may underlie, at least in part, a resistance-sparing effect of mVNR.

The median PFS was 22.1 months and, after a median follow-up of 27 months, 73% (95CI 47–100%).

Only three patients received full DEVEC, all of whom encountered severe infections or febrile neutropenia, prompting a switch to DEVEC-light. Omitting etoposide still resulted in high response rates while reducing hematologic toxicity. This finding is consistent with prior observations that etoposide is the main driver of myelosuppression in metronomic regimens [31]. Notably, R-DEVEC-light was very well tolerated in patients who stopped lenalidomide due to toxicity, as well as in patients with severe kidney and hepatic impairments.

The integration of rituximab with vinorelbine in the maintenance phase also likely contributed to the high and durable CR rates and PFS in this series of heavily treated subjects [39].

Interestingly, long-term vinorelbine maintenance is feasible, with some patients continuing it beyond 20–100+ cycles. Currently, there is no data to support whether patients in remission benefit from indefinite maintenance therapy with vinorelbine. Indeed, one multiple-relapsed patient who discontinued vinorelbine after 12 months of maintenance is still in continuous CR at 84 months from the start of treatment (Figure 1).

Recently, several trials aiming at ameliorating the outcome of rrFL were published. The phase III randomized inMIND study showed that after a median follow-up of 14.1 months, the addition of tafasitamab to R^2^ resulted in an estimated progression-free survival (PFS) of 22.4 months compared to 13.9 months of the R^2^ arm [10]. Obinutuzumab–bendamustine demonstrated a PFS advantage over bendamustine alone (26 vs. 14 months) but is not ideal for frail patients, due to increased cytopenias and infectious complications [40]. PI3K inhibitors such as idelalisib, duvelisib, and copanlisib yielded ORR of 47–59% but were characterized by severe immune-mediated toxicities and deaths, which ultimately led to restricted use or withdrawal in several regions [41,42,43]. Zanubrutinib, a next-generation BTK inhibitor, in combination with obinutuzumab, was recently approved by EMA and FDA for the treatment of patients with rrFL who received at least two prior lines of therapy, based on results of the ROSEWOOD study [44]. Zanubrutinib plus obinutuzumab showed favorable outcomes compared to obinutuzumab monotherapy, with ORRs of 69% versus 46%, and median PFSs of 28 months and 10.4 months, respectively. Importantly, patients receiving the combination experienced lower rates of pyrexia and infusion-related reactions compared with those treated with obinutuzumab alone [44]. Tazemetostat, an oral EZH2 inhibitor, showed an ORR of 69% in EZH2-mutated and 35% in wild-type rrFL with excellent tolerability [45]. However, its approval is limited to selected molecular subgroups (i.e., at least two prior lines of therapy and documented EZH2 status). Tazemetostat in combination with R^2^ for rrFL is being evaluated in the phase 3 part of the Symphony-1 study [46].

Histone deacetylase (HDAC) inhibitors, including abexinostat, vorinostat, and mocetinostat, have also shown clinical activity in relapsed FL, with variable response rates across studies [47,48,49].

Following the success of anti-CD20 therapies, several new immunotherapy approaches are under investigation, including bispecific antibodies, antibody–drug conjugates, immune-checkpoint inhibitors, and CAR-T-cell therapy.

The combination of the anti–PD-1 monoclonal antibody pembrolizumab with rituximab demonstrated clinical activity in patients with rrFL, achieving an ORR of 67% and a CRR of 50%. However, despite these encouraging results, the study was limited to patients who were sensitive to rituximab, and the efficacy of this regimen may be reduced in individuals with rituximab-refractory disease [50].

CAR-T cell therapy (i.e., tisagenlecleucel and axicabtagene ciloleucel), and bispecific antibodies (i.e., mosunetuzumab, glofitamab, and epcoritamab) achieved impressive response rates and prolonged PFS even in multiply relapsed FL [51,52,53]. Epcoritamab simultaneously binds the B-cell antigen CD20 and the T-cell antigen CD3, leading to T-cell-mediated cytotoxicity of CD20-expressing malignant B-cells, approved in numerous countries as monotherapy for rrFL after two or more lines of therapy [52]. Notably, the combination of epcoritamab, rituximab, and lenalidomide has been recently approved by the FDA [54]. More recently, the combination of epcoritamab plus R^2^ significantly increased response rates and lowered the risk of disease progression or death compared with R^2^ alone in individuals with rrFL. Grade 3 or higher adverse events were more common with the triplet than with R^2^ but remained manageable [55].

Antibody–drug conjugates have further expanded treatment options by delivering targeted cytotoxic payloads to malignant B-cells. Polatuzumab vedotin, by targeting the CD79b component of the B-cell receptor (BCR) and releasing the microtubule inhibitor monomethyl auristatin E, in combination with rituximab, achieves an ORR of 70%, including a 45% complete response rate [56]. However, very elderly or frail patients, due to comorbidities, risk of prolonged immune-suppression, logistical barriers (for CAR-T cell therapy), eligibility restrictions, and costs, may be ineligible for this treatment.

Older patients exhibit changes in tumor–host interactions and a higher burden of comorbidities, leading to alterations in pharmacokinetics and pharmacodynamics that may contribute to poorer outcomes in this population [57]. Moreover, aging is associated with progressive impairment of DNA damage–repair mechanisms and a reduction in both cell-mediated and humoral immune response [58]. In the era of rituximab-based therapy, elevated lymphoma-associated macrophage levels have been linked to better outcomes at both initial diagnosis and disease relapse, probably reflecting enhanced antibody-dependent cellular cytotoxicity [59]. Conversely, younger patients tend to have a lower relative proportion of lymphoma-associated macrophages. Nonetheless, if administered at the appropriate dose intensity, chemotherapy regimens in elderly subjects can yield response rates equivalent to those achieved in younger cohorts [58].

Although T-cell–engaging therapies have achieved unprecedented success, the outcomes of patients treated with the mCHEMO schedule remain encouraging, as approximately 50% of these patients had previously discontinued lenalidomide (Table 1). Furthermore, most deaths were attributable to comorbidities rather than lymphoma progression. The toxicity profile in this cohort was more favorable than in our previous studies of DLBCL and peripheral T-cell lymphoma (PTCL), who received DEVEC without omission of etoposide [28,31]. This reduction in adverse effects is likely due to the omission of etoposide for the majority of patients; significantly, this modification does not appear to compromise clinical outcomes in indolent follicular lymphoma. Indeed, DEVEC-light was very active as the ORR was 84%. Notably, all three heavily pretreated subjects, who started DEVEC, had to stop etoposide for excessive toxicity between cycles 2 and 3.

Neutropenia (31%) was the most common adverse grade ≥3 event. Two subjects died during treatment, and one 94-year-old patient died six months after the end of treatment for infection. Notably, only one death results from histologic transformation to high-grade lymphoma. This low rate of disease-related mortality aligns with our previous DEVEC experiences in elderly patients with aggressive NHL, while most deaths were not considered, at least directly, related to treatment toxicity [29]. Conversely, they predominantly reflect the intrinsic vulnerability of this population.

The course of the two patients who relapsed after DEVEC-light but subsequently achieved CR highlights the resistance-sparing feature of metronomic therapy: it preserves sensitivity to later conventional treatments (i.e., R^2^ and obinutuzumab-CVP). These observations suggest that mCHEMO may also be an effective bridging or disease-stabilizing approach without inducing cross-resistance to later lines of therapy, consistent with its ability to target angiogenesis, cancer stem cells, and modulate the immune system [60,61,62]. Thus, patients who eventually relapse after mCHEMO may retain responsiveness to standard agents, including immunomodulators, anti-CD20 monoclonal antibodies, and conventional immune-chemotherapy [63].

Nowadays, diagnoses of relapsed disease in patients over 75 years have become very common. Given the increased susceptibility to treatment-related toxicity and the impaired immune response in older patients [57], therapeutic decisions in this population should be made with careful evaluation and expert guidance.

Indeed, this study extends our previous DEVEC work in DLBCL and PTCL [28,29,31], which are aggressive diseases with a different biology from FL. In fact, the activity of mCHEMO in the lenalidomide-ineligible population, the maintenance-driven deepening of response, is a novel finding. As far as we know, the available evidence on mCHEMO in FL is very limited; however, it highlights the promising activity of this approach [64,65]. Our experience shows this metronomic regimen, together with the flexibility to adjust dosing based on individual tolerance, represents a practical option for elderly patients.

## 5. Conclusions

Metronomic R-DEVEC-light provides a feasible, low-intensity, and home-based treatment option for frail, heavily pretreated patients with rrFL. Owing to its favorable safety profile, oral administration, cost-effectiveness, and lower risk of inducing treatment resistance, this regimen can be considered a rational option in later treatment lines for patients who are unfit for standard immuno-chemotherapy, ineligible for T-cell-engaging therapies, and in whom lenalidomide is contraindicated or poorly tolerated. In this clinically challenging population, R-DEVEC-light may serve as a pragmatic disease-control strategy, offering meaningful responses with manageable toxicity when therapeutic alternatives are limited.

Additional studies are clearly warranted to validate the clinical relevance of DEVEC-light in rrFL. Finally, considering the accessibility to successful non-chemo-immunotherapy approaches [3], pharmacological investigations assessing the synergistic potential of DEVEC-light in combination with targeted therapies may further improve outcomes while preserving tolerability in patients with rrFL.

## Figures and Tables

**Table 1:** Clinical and epidemiological features of 13 patients with Follicular Lymphoma.

Characteristics	Patients (*n* = 13)
Age (median, range)	77 years (58–92)
Sex, male (%)	5/13 (38%)
POD24	7/11 (64%)
Previous lines (median, range)	3 (* 0–5)
Lenalidomide treatment	Discontinued for toxicity 6/13 Controindicated 3/13 Not available 3/13 Refractory 1/13
FLIPI before mCHEMO median (range)	3 (2–4)
mCHEMO cycles (median, range)	
Induction	6
Maintenance	12 (0–112)
ORR	12/13 (92%)
CRR	9/13 (69%)
PD	2/13 (16.4%)

POD24: Progression of disease after first-line systemic therapy within 24 months from the end of induction; mCHEMO: metronomic chemotherapy; ORR: overall response rate; CRR: Complete remission rate; PD: progressive disease. * Two patients who started Rituximab–lenalidomide (R^2^) upfront, discontinued R^2^ during cycle 1 and 2, respectively.

## Data Availability

The data are available at the Hematology Unit, Fondazione Policlinico Tor Vergata (Dr. M.C.Cox).

## Abbreviations

The following abbreviations are used in this manuscript:

FL	Follicular lymphoma
rrFL	Relapsed or refractory follicular lymphoma
NHL	Non-Hodgkin lymphoma
DLBCL	Diffuse large B-cell lymphoma
PTCL	Peripheral T-cell Lymphoma
mCHEMO	Metronomic chemotherapy
CGA	Comprehensive geriatric assessment
CAR-T	Chimeric antigen receptor T-cell therapies
R-DEVEC	Deltacortene^®^ [prednisone], etoposide, vinorelbine, and cyclophosphamide, + rituximab
POD24	Progression of disease within 24 months
OS	Overall survival
PFS	Progression-free survival
CR	Complete remission
PR	Partial response
PD	Progressive disease
ORR	Objective response rate
CRR	Complete remission rate
R^2^	Rituximab + lenalidomide
CVP	Cyclophosphamide, prednisolone, and vincristine
PI3K	Phosphoinositide 3-kinase
EZH2	Enhancer of zeste homolog 2
